# EDAM: an ontology of bioinformatics operations, types of data and identifiers, topics and formats

**DOI:** 10.1093/bioinformatics/btt113

**Published:** 2013-03-11

**Authors:** Jon Ison, Matúš Kalaš, Inge Jonassen, Dan Bolser, Mahmut Uludag, Hamish McWilliam, James Malone, Rodrigo Lopez, Steve Pettifer, Peter Rice

**Affiliations:** ^1^EMBL European Bioinformatics Institute, Hinxton, Cambridge CB10 1SD, UK, ^2^Computational Biology Unit, Uni Computing, 5008 Bergen, Norway, ^3^Department of Informatics, University of Bergen, 5008 Bergen, Norway and ^4^School of Computer Science, The University of Manchester, Manchester, M13 9PL, UK

## Abstract

**Motivation:** Advancing the search, publication and integration of bioinformatics tools and resources demands consistent machine-understandable descriptions. A comprehensive ontology allowing such descriptions is therefore required.

**Results:** EDAM is an ontology of bioinformatics operations (tool or workflow functions), types of data and identifiers, application domains and data formats. EDAM supports semantic annotation of diverse entities such as Web services, databases, programmatic libraries, standalone tools, interactive applications, data schemas, datasets and publications within bioinformatics. EDAM applies to organizing and finding suitable tools and data and to automating their integration into complex applications or workflows. It includes over 2200 defined concepts and has successfully been used for annotations and implementations.

**Availability:** The latest stable version of EDAM is available in OWL format from http://edamontology.org/EDAM.owl and in OBO format from http://edamontology.org/EDAM.obo. It can be viewed online at the NCBO BioPortal and the EBI Ontology Lookup Service. For documentation and license please refer to http://edamontology.org. This article describes version 1.2 available at http://edamontology.org/EDAM_1.2.owl.

**Contact: **jison@ebi.ac.uk

## 1 INTRODUCTION

The number and diversity of bioinformatics tools, including data resources, grows vastly. To aid users in finding, comparing, selecting and integrating tools into workflows or workbenches, it is important having the tools consistently described with respect to a number of categories. These include their application domain (e.g. protein structure, metagenomics), function (e.g. alignment construction), type of input and output data (e.g. accession, feature record) and available formats of the data (e.g. FASTQ, PDB format). In the absence of accepted standards for such tool descriptions, the categorization of tools has been left to providers of tool catalogues or workbenches. In this undesired situation, tools have to be described again every time they are integrated into a new framework. Not only duplicating efforts, this also leads to fragmented descriptions and inconsistent categorization.

We present EDAM, an ontology of bioinformatics operations, types of data and identifiers, data formats and topics. Its name originates from ‘EMBRACE Data And Methods’, as it was initiated by the EMBRACE project (Pettifer *et al.*, 2010). Its primary goal is as a means of creating coherent, machine-understandable annotations for use within resource catalogues [such as BioCatalogue (Bhagat *et al.*, 2010) or myExperiment (Goble *et al.*, 2010)], information standards (such as BioDBCore, Gaudet *et al.*, 2011), Web services (http://www.w3.org/standards/webofservices), collaborative infrastructures (such as Elixir, http://www.elixir-europe.org), tool collections [e.g. Bio-Linux (Field *et al.*, 2006) and Debian Med (Möller *et al.*, 2010)] and integrated workbenches (e.g. Galaxy, Goecks *et al.*, 2010). EDAM is also intended to complement standards for data exchange, enrich provenance metadata, offer a shared markup vocabulary for bioinformatics data on the Semantic Web and aid text mining by defining interrelated terms and synonyms. In addition, EDAM must be conveniently usable by annotators and tool users ranging from programmers to lab biologists.

To ensure good coverage of common concepts, numerous tools and databases have been semantically annotated with EDAM. Functionality that makes use of EDAM annotations has been implemented in a set of representative frameworks: a suite of bioinformatics tools (EMBOSS, Rice *et al.*, 2000), an integrated workbench for data sharing and analysis (eSysbio, http://esysbio.org), and a workflow system (Bio-jETI, Lamprecht *et al.*, 2011), thus testing the usability of EDAM.

### 1.1 Related work within bioinformatics

The field of data and resource integration within bioinformatics has received significant attention over the past decade, with standardization efforts falling into three categories: information standards, data models and ontologies.

**Information standards** such as those unified under MIBBI (Minimum Information about a Biomedical or Biological Investigation, Taylor *et al.*, 2008) define what information should be recorded when reporting scientific experiments. For example, MIGS (Minimum Information about a Genome Sequence) and related MIxS standards require specific metadata for genomic sequences (Field *et al.*, 2008; Yilmaz *et al.*, 2011).

**Data models**, schemas or exchange formats define structures for data representation and enable convenient sharing between tools. Various data models have been developed, ranging from specific textual or binary formats (e.g. SAM and BAM, Li *et al.*, 2009) to formal machine-understandable schemas. XML Schema-based approaches include BioXSD for basic types of data in bioinformatics (Kalaš *et al.*, 2010), and more specialized formats such as phyloXML and NeXML for phylogenetics (Han and Zmasek, 2009; Vos *et al.*, 2011) or GCDML for MIGS-compliant metadata (Kottmann *et al.*, 2008). Alternatively, data models can be defined using an ontology language, as exemplified by the BioMoby Object Ontology defining XML exchange formats within the BioMoby framework (Wilkinson *et al.*, 2008), and the BioPAX exchange format for pathway data (Demir *et al.*, 2010).

**Ontologies** can be used to define data models, but more commonly they define collections of interrelated items. These range from informal lists such as those used to categorize the articles in journals, through Nucleic Acids Research’s hierarchies of database and Web-server categories (Benson, 2011; Galperin and Fernández-Suárez, 2012), to formal ontologies establishing commonly understood meaning and relations of subjects in focus. Examples are the widely used Gene Ontology (GO) of biological processes, molecular functions and cellular components (Ashburner *et al.*, 2000), the Sequence Ontology (SO) of nucleic acid and protein features (Eilbeck *et al.*, 2005) or the Comparative Data Analysis Ontology (CDAO) for phylogenetics (Prosdocimi *et al.*, 2009).

The myGrid ontology (Wolstencroft *et al.*, 2007) was developed for annotating bioinformatics tools with their types of interface, operations, types of input/output data and formats. In addition, it listed some concrete algorithms, databases, types of database records and identifiers. The myGrid ontology is no longer maintained, but it served as a starting point for the development of EDAM.

### 1.2 Other related work

Several projects outside the life sciences are relevant to the objectives of this work. DOAP (Description Of A Project, https://github.com/edumbill/doap/wiki) is a vocabulary of domain-agnostic metadata attributes of a software project, such as its programming language, operating system, developer or homepage. The standard Semantic Web vocabularies such as RDFS (http://www.w3.org/TR/rdf-schema) and Dublin Core (http://dublincore.org) include basic types of data for describing digital artefacts, e.g. label, comment or identifier. OWL-S (Martin *et al.*, 2004) and WSMO (Roman *et al.*, 2005) ontologies aim at enabling automated discovery and composition of Web services, independent of an application domain. Several efforts have developed for preservation of information and digital media (including software), for example the ISO OAIS Reference Model (ISO, 2002), the PRONOM file-format registry and associated tools (Brody *et al.*, 2007) and the PREMIS metadata model, vocabulary and format (Dappert and Enders, 2010). The Wf4Ever project focusses on preservation of scientific workflows (http://wf4ever-project.org).

Ontologies for describing data-mining experiments such as DMOP (http://www.dmo-foundry.org/DMOP) include methods and parameters used in data mining, both within and outside of life sciences. OntoDT (http://kt.ijs.si/panovp/doku.php?id=ontodt) comprises programming datatypes and data structures. Some ontologies have been developed to comprehensively enumerate diverse domain-unspecific entities. Notable among these are Cyc (Lenat, 1995) and the Suggested Upper Merged Ontology (SUMO, Niles and Pease, 2001).

### 1.3 Scope for EDAM

In spite of the breadth and diversity of the existing ontologies, none provides a comprehensive means of classifying bioinformatics operations, types of data and identifiers, data formats and topics in a way that is suitable for large-scale semantic annotations and categorization of bioinformatics resources. Among previous ontology projects within bioinformatics, the myGrid ontology had the most similar scope, but is no longer maintained. On the other hand, multiple vocabularies outside of life sciences aim at describing tools and data resources, but they do not include the necessary bioinformatics-specific concepts. EDAM was developed to fill this niche.

The rest of the article is organized as follows: the *Methods* section describes the main design principles used in EDAM. *Results* describe EDAM, the annotations with EDAM and the implementation projects that adopted EDAM. *Conclusion* summarizes the article.

## 2 METHODS

The main design principles of EDAM are *relevance* to its target applications, convenient *usability* for annotators and users of the annotations and efficient *maintainability* by its developers.

To ensure **relevance**, EDAM has to comprehensively cover the common bioinformatics concepts. To achieve this, numerous resources were analysed and used as sources of concepts. The myGrid ontology served as a starting point. Collections of tools were analysed, including Web services from the EMBRACE registry (Pettifer *et al.*, 2009), the EMBOSS suite and the BioMoby Service Ontology. Common bioinformatics data formats and the BioMoby Object Ontology served as sources of types of data and formats. The Nucleic Acids Research’s database and Web-server catalogues, as well as classifications within bioinformatics journals and conferences were used as sources of topics. Semantic annotations with EDAM and the implementations using EDAM, done in parallel with the EDAM development, provided valuable feedback.

Heuristics for ensuring that EDAM remains broadly applicable include logical consistency, clear semantic scope, well-defined interfaces with other ontologies and being open to future developments in collaboration with the community.

EDAM has to be conveniently **usable** by humans for the purposes of annotation and search. We have therefore avoided excessively broad or deep branches and have orientated the ontology around the small number of ‘orthogonal axes’ (sub-ontologies), each with readily understood meaning.

To keep EDAM **maintainable**, agile software development methods are used. This ensures that changes are delivered with good response time using limited resources and yielding consistent results. For example, relations between concepts are explicitly defined only in one direction, to minimize the possibility for inconsistencies and to ease maintenance.

EDAM’s design is not based on any metaphysical doctrine, but that does not mean that it is based on bad or no philosophy. EDAM is founded on logic, and on relevance and utility to the bioinformatics community. This is in accordance with Lord and Stevens (2010), Merrill (2010, 2011) and Rzhetsky and Evans (2011) that all indicate, using separate sets of arguments, that it is the relevance of scientific ontologies with respect to their practical applications that is more important than an imposed metaphysical ideology. EDAM *concepts* are not concepts existing only in minds of the EDAM authors, but common notions shared within the bioinformatics community.

EDAM follows the accepted OBO Foundry principles (Open Biological and Biomedical Ontologies Foundry, http://www.obofoundry.org/wiki/index.php/Category:Accepted, Ashburner *et al.*, 2003). The scope is clearly focussed and unique. All concepts include definitions. These are concise, sufficient to delineate the concepts, but avoiding details that would be irrelevant to target applications. EDAM syntax and logical structure has been validated by OWL reasoners in Protégé (http://protege.stanford.edu).

EDAM follows to some extent also the candidate OBO Foundry principles under discussion (http://www.obofoundry.org/wiki/index.php/Category:Discussion), with a few exceptions owing to the usability, maintainability or coherence requirements. For example, terms are capitalized for aesthetic reasons and faster recognition. In some places, specialization of multiple generic concepts is logically correct and necessary for usability, such as in *Structure alignment* being both an *Alignment* and *Structure*.

Some mostly higher-level concepts are related to generic Semantic Web vocabularies or to higher-level concepts in specialized ontologies with different focus than EDAM: e.g. RDFS, Dublin Core, DOAP, DMOP, BRO (Tenenbaum *et al.*, 2011) or MeSH (Nelson, 2009). This applies also to ontologies under development: the Semanticscience Integrated Ontology (SIO, http://code.google.com/p/semanticscience/wiki/SIO), Web Service Interaction Ontology (WSIO, http://wsio.org) and SoftWare Ontology (SWO, http://theswo.sourceforge.net). Such concepts are linked from EDAM. Additionally, in the case of SWO, the bioinformatics-specific concepts of EDAM are included via OWL import. The higher-level concepts in EDAM also reference concepts in multiple upper ontologies: DOLCE (Gangemi *et al.*, 2002), BioTop (Beisswanger *et al.*, 2008), GFO and GFO-Bio (Hoehndorf *et al.*, 2008), BFO (Grenon *et al.*, 2004) and SUMO. EDAM may thus be usable in a variety of future semantic-integration scenarios. In addition, some concepts in EDAM include links to other scientific ontologies with different ‘axes’ of meaning or with more detail. These include SO, CDAO, GO and ChEBI (Degtyarenko *et al.*, 2008). EDAM relations explicitly reference the relations defined in the Relation Ontology (Smith *et al.*, 2005), IAO (http://code.google.com/p/information-artifact-ontology) and OBI (Smith *et al.*, 2007). For example, *has input* points to *has_specified_input* in OBI and *has topic* points to *is about* in IAO, via links with comments explaining the differences in meanings.

EDAM has been iteratively developed yielding on average four versions released per year (in the course of the last 4 years), resulting in the current version 1.2. Concept URIs and IDs persist between EDAM versions. The name, definition, relations and other properties may change; nonetheless a given URI (ID) will remain fundamentally true to the original concept. Concepts may be deprecated on the release of a new version, but they persist, with their original ID and URI. Concept URIs do not contain a version, so semantic annotations remain valid while EDAM evolves, without an immediate need for update. Deprecated concepts indicate a replacement (via *replaced_by*), or one or more suggestions (via *consider*). EDAM will continue evolving, but future versions should not be a fundamental departure from the established scope, principles and architecture.

## 3 RESULTS

### 3.1 The EDAM ontology

**Fig. 1.**
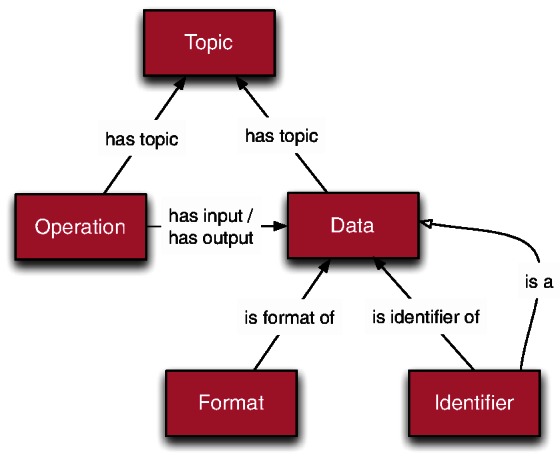
Organization of the main EDAM sub-ontologies and the relations explicitly maintained between EDAM concepts

EDAM consists of four main sub-ontologies rooted in the top level of its hierarchy: ***Operation***, ***Data***, ***Topic*** and ***Format*** (Table 1 and Fig. 1). A fifth distinguishable sub-ontology is ***Identifier*** rooted under ***Data***. ***Operation*** concepts denote what function a tool provides or how a piece of data was created. ***Data*** concepts can denote what data a tool consumes and produces, what a dataset contains or what type of data an attribute is. Focus lies on the types of data (the content) and not on datatypes (the runtime representation defined in a programming language). ***Identifier*** sub-ontology comprehensively catalogues the types of life-scientific identifiers in common use. ***Topic*** contains coarse-grained domains of a wide range of bioinformatics resources. Finally, ***Format*** catalogues the commonly used data formats used by bioinformatics tools and data.

**Table 1.** The main EDAM sub-ontologies

**Sub-ontology**	**Definition**	**Scope within EDAM**	**Examples of terms**	**Number of concepts**
Operation	A function that processes a set of inputs and results in a set of outputs, or associates arguments (inputs) with values (outputs). Special cases are: (a) An operation that consumes no input (has no input arguments). Such operation is either a constant function, or an operation depending only on the underlying state. (b) An operation that may modify the underlying state but has no output. (c) The singular-case operation with no input or output, that still may modify the underlying state	Singular, bioinformatics-specific operations that are functions of tools, workflows or scripts, or can be performed manually	RNA structure prediction Protein docking Data retrieval	558
Data	Information, represented in an information artefact (data record) that is ‘understandable’ by dedicated computational tools that can use the data as input or produce it as output	Types of data that are relevant in bioinformatics, commonly used as inputs, outputs or intermediate data of analyses, or provided by databases and portals	Sequence Sequence record Phylogenetic tree UniProt accession	1140
Identifier (under Data)	A text token, number or something else that identifies an entity, but which may not be persistent (stable) or unique (the same identifier may identify multiple things)	Types of identifiers that identify biological or computational entities; including resource-specific data accessions. Several identifier concepts in EDAM include regular expressions and examples	UniProt accession EC number	528
Topic	A category denoting a rather broad domain or field of interest, of study, application, work, data or technology. Topics have no clearly defined borders between each other	Application domains of bioinformatics tools and resources; topics of research, studies or analyses; approaches, techniques and paradigms within—or directly related to—Bioinformatics	Sequence analysis Phylogenetics ontology	209
Format	A defined way or layout of representing and structuring data in a computer file, blob, string, message or elsewhere.	Data formats commonly used in—and specific to—Bioinformatics. Many format concepts in EDAM include references to their definition and documentation	BAM GVF SBML	347

*Note*: The EDAM sub-ontologies contain common concepts specific—or directly related—to bioinformatics.

Twelve types of relations are defined in EDAM (Table 2). Five of these are maintained explicitly, in addition to the standard generalization relation *is a*. All types of relations are applicable to semantic annotation of relevant entities.

**Table 2.** Types of relations defined in EDAM

**Relation**	**Inverse**	**Maintained in EDAM**	**Example**
*Has input*	*Is input of*	***Operation*** has input ***Data***	*Sequence annotation* has input *Sequence record*
*Has output*	*Is output of*	***Operation*** has output ***Data***	*RNA structure prediction* has output *RNA structure record*
*Has topic*	*Is topic of*	***Operation*** or ***Data*** has topic ***Topic***	*Phylogenetic tree* has topic *Phylogenetics*
*Has format*	*Is format of*	***Format*** is format of ***Data***	*CHP* is format of *Processed microarray data*
*Has identifier*	*Is identifier of*	***Identifier*** is identifier of ***Data***	*InterPro accession* is identifier of *Protein signature*
*Has function*	*Is function of*	Not between EDAM concepts	A tool has function *Sequence assembly*

*Note*: Definitions, domains and ranges are present in the EDAM.*owl* file. EDAM relations apply between concepts and/or annotated entities.

Concepts are identified by global URIs of the form http://edamontology.org/<subontology>_<localId>. The local IDs have four digits. In the OBO-format version of EDAM, concept identifiers have form *EDAM_*(*subontology*):(*localId*). For example, *Sequence record* is identified by http://edamontology.org/data_0849 or *EDAM_data:0849*. Relation types and additional concept properties are identified by http://edamontology.org/<id> or *EDAM*:(*id*), such as http://edamontology.org/has_function and *EDAM:has_function*. EDAM URIs follow the good practices (http://www.w3.org/Provider/Style/URI). They are stable, easily maintainable, HTTP, dereferenceable, simple and concise. The concise form of the EDAM URIs is convenient for annotations and for use on the Semantic Web, and less prone to typos. Different representations of EDAM are available via HTTP content negotiation: http://edamontology.org redirects to http://edamontology.org/page, http://edamontology.org/EDAM.owl, http://edamontology.org/EDAM.obo or http://edamontology.org/EDAM.uris, depending on the requested media type. URIs of single EDAM concepts either redirect to a dedicated Web page in the NCBO BioPortal, or return a machine-understandable representation (full *EDAM.owl* is returned in order to maintain context). A *?format = *query can be used as an alternative to content negotiation.

Concept declarations in EDAM contain a primary label (the recommended term), synonyms, definition, relations to other concepts in EDAM and links to related concepts in other resources. Some concepts have additional information. *Regular expression* constrains allowed values of types of identifiers (mostly accessions) and is useful for validation of inputs to tools. As examples, EMBOSS will in the future use regular expressions from EDAM to validate identifiers before requesting the corresponding data, and BioXSD will include accession types generated from EDAM, with the constraining patterns. *Example* lists one or more valid examples (among the identifiers). *Documentation* includes a URL within a ***Format*** concept pointing to its documentation. *Created in* states which version of EDAM a concept was added in. *Obsolete since* states the version since which an obsolete concept has been deprecated.

The latest stable version of EDAM can be downloaded in OWL format from http://edamontology.org/EDAM.owl and in OBO format from http://edamontology.org/EDAM.obo. OWL in RDF/XML is the primary format EDAM is maintained in, while the OBO version lacks some minor details. EDAM can be browsed online at the NCBO BioPortal (Noy *et al.*, 2009) or EBI’s Ontology Lookup Service (OLS, Côté *et al.*, 2010). Programmatic access to EDAM is provided by a suite of tools in EMBOSS and by the NCBO Web services.

### 3.2 Semantic annotation with EDAM

There are two main approaches to annotation of tools. (i) Tools represented by a standardized information artefact can contain the annotations in these descriptions. This applies to Web services with their WSDL files and to XML Schemas for which there is a common standard for semantic annotation: SAWSDL (Kopecky *et al.*, 2007). Within the SADI framework (Wilkinson *et al.*, 2011), services are described in dedicated RDF documents using the structure defined in The Moby-myGrid Service Ontology (http://www.mygrid.org.uk/mygrid-moby-service). For scripts represented by their source code, an annotation format is promisingly emerging (Kallio *et al.*, 2011). Annotations in standard descriptions of tools are provided and maintained by providers of the tools, and are independent of context and catalogues. Therefore these tools do not need to be annotated again when integrated into a new framework. (ii) Annotations can be provided, stored and maintained in dedicated catalogues, in proprietary formats. This option applies to all kinds of resources.

All tools in the **EMBOSS** toolkit for bioinformatics analyses (Rice *et al.*, 2000) have their topics, operations, inputs and outputs annotated with EDAM. These annotations are present in each Application Command Definition (ACD) file, which describes a tool’s command-line interface. The ACD files can be downloaded as part of the EMBOSS and associated EMBASSY packages (ftp://emboss.open-bio.org/pub/EMBOSS).

**Fig. 2.**
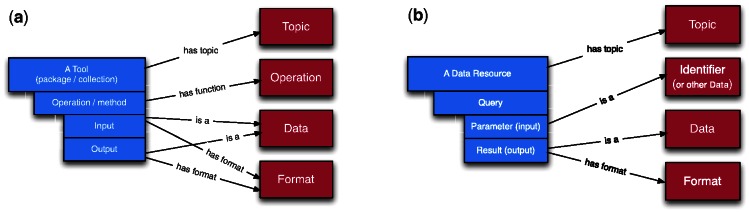
Sketches of information models for semantic annotations with EDAM. (**a**) A model for annotations of tools corresponding to the SAWSDL standard (Kopecky *et al.*, 2007). Standardizing an information model of tool metadata is, at least so far, out of scope of EDAM. (**b**) A similar model for annotations of data resources, used within DRCAT. Note that a query has always (implicitly) the function of *Data retrieval*. Defining an information standard for database metadata is within scope of the BioDBCore initiative (Gaudet *et al.*, 2011)

**Web services** from various providers were annotated with EDAM, either within the EMBRACE project (Pettifer *et al.*, 2010) or with help of public workshops and tutorials. These include, for example, the iHOP Web service (Fernández *et al.*, 2007, http://ws.bioinfo.cnio.es/iHOP/#EMBRACE), WSDbfetch (http://www.ebi.ac.uk/ws/wsdl/WSDBFetchDoclitServerService.wsdl) and services provided by the Computational Biology Unit in Bergen (http://cbu.bioinfo.no/wsdl). Annotations of Web services use the simple information model recommended by EMBRACE and SAWSDL (Fig. 2a). Experience has shown that using this EDAM-EMBRACE-SAWSDL approach, providers can annotate their services with minor effort. As more applications make use of annotations with EDAM, the annotation effort results in better visibility and usability of the provided tools or resources.

In **BioXSD**, the XML format of basic bioinformatics types of data (Kalaš *et al.*, 2010), the type definitions and the data parts are annotated with ***Data*** sub-ontology, using SAWSDL. This gives BioXSD types interoperable semantics and they can serve as pre-annotated building blocks for tool interfaces. Naturally, the complexType-s in BioXSD are in addition annotated as having format *BioXSD*. The annotations can be viewed in the BioXSD Schema (http://bioxsd.org/BioXSD-1.1.xsd).

**DRCAT**, the Data Resource CATalogue (http://drcat.sourceforge.net), collates metadata on bioinformatics data resources including databases, data warehouses, portals and taxonomies. A DRCAT entry includes information such as resource identifier, name, taxon, URL and, importantly, URL-based queries. Annotation with EDAM denotes topics of the resources, types of data provided, query parameters and output formats. DRCAT is a work in progress but the current version includes 655 entries, 521 query lines and 2147 EDAM annotations. The model of EDAM annotations in DRCAT is sketched in Figure 2b and examples can be viewed at http://drcat.sourceforge.net/#3.

**SEQanswers** portal provides a wiki catalogue of bioinformatics tools, with focus on high-throughput sequencing analysis (Li *et al.*, 2012, http://seqanswers.com/wiki/Software). Where applicable, the SEQanswers methods and domains are represented by EDAM concepts (mostly from ***Operation*** and ***Topic***). Input and output formats will be represented by EDAM concepts in the near future. Currently the mapping to EDAM is done by matching tags to concept labels; however, a complete manual mapping that includes synonyms has been performed and will be reflected in due course. Use of EDAM within SEQanswers results in more interoperable descriptions of the collated tools, and allows searching and filtering by the concepts.

### 3.3 Implementations using EDAM

In addition to having all its tools annotated, the **EMBOSS** suite provides comprehensive tooling for EDAM-driven queries of the tools and DRCAT (http://emboss.open-bio.org/rel/rel6/apps/ontology_edam_group.html). This includes finding data resources by the data or formats served, or by identifiers used in queries, finding all EMBOSS tools by EDAM data (input and/or output, and other parameters), operation or topic and finding EDAM concepts by id, name, definition or which have certain relations defined. The concept hierarchy is taken into account.

Applicability of EDAM to integrative workbenches has been validated by implementations in eSysbio (http://esysbio.org) and Bio-jETI (Lamprecht *et al.*, 2011).

**eSysbio** is a prototype online workbench for analysing bioinformatics data using shared or private Web services and R scripts, and for sharing the data and tools among users. eSysbio uses EDAM ***Data*** and ***Format*** to decide how to handle data uploaded by users or produced by workflows. EDAM annotation enables adequate visualization and search among the data stored in the system. For example, a data item, annotated as an *Alignment* and a supported ***Format***, will be open with the Jalview editor (Waterhouse *et al.*, 2009). The current version of eSysbio uses a limited subset of EDAM for static navigation, without taking into account the relations other than the closure of *is format of*. It allows grouping and filtering of data by their type, and sorting by type and format. eSysbio may use the entire EDAM and its semantics in the future. This can include the ***Operation*** and ***Topic*** sub-ontologies for categorization and search among available Web services, scripts and workflows, and as part of the provenance metadata for derived data items.

**Bio-jETI** is a system for design, model checking and execution of bioinformatics workflows. Bio-jETI uses EDAM ***Operation***, ***Data*** and ***Format*** annotations of EMBOSS and other tools to enable automatic composition of workflows, according to formal specifications defining what the workflow is supposed to compute (expressed using EDAM, too). The automated reasoning software in Bio-jETI saves from matching different interfaces and formats manually, by suggesting one or more alternative workflows fulfilling the task. This has been shown to work for tasks that can be easily defined. Details about the use of EDAM in Bio-jETI can be found in Lamprecht *et al.* (2011).

## 4 CONCLUSION

We have presented EDAM, the ontology that applies to semantic annotation of tool functions, types of data and identifiers, data formats and the domains of diverse resources within bioinformatics. The development of EDAM has been application driven, but EDAM is not application specific. Its usability has been tested by annotating a multitude of tools and data resources. EDAM’s applicability to searching, categorizing and automatic handling of resources has been validated by implementations in eSysbio, Bio-jETI and EMBOSS, demonstrating its relevance to resource catalogues, tool libraries and integrative workbenches within bioinformatics. EDAM is also relevant to data provenance, text mining and the Semantic Web. Applicability of EDAM as one of the markup vocabularies for bioinformatics data in RDF was tested at the fourth BioHackathon in Kyoto (example at https://github.com/dbcls/bh11/wiki/BioXSD-sequence-record-in-RDF).

EDAM does not try to cover all aspects of computational biology. It focusses purely on the semantic ‘axes’ delineated by its four main sub-ontologies: ***Operation***, ***Data*** (including ***Identifier***), ***Topic*** and ***Format***, in which it targets the common bioinformatics concepts, especially those reused in multiple contexts. Concepts from distinct EDAM sub-ontologies are related by a few basic relations in addition to generalization (*is a*) which constitutes the basic hierarchy. EDAM does not define the aggregation relation (*is part of*, *has part*, *has a* or *contains*). What particular computational steps are done inside an operation is defined by a particular algorithm or a workflow, and it may vary between different implementations of the same operation. In the same way for a type of data, what parts it must or may contain is defined by a concrete data model or format, an information standard or reporting requirement. The included parts of data, both mandatory and optional, differ between different formats of the same type of data. While not defining data and operation parts universally, EDAM does offer concepts for annotating the parts of a particular data format or dataset, and concepts for annotating the steps of a particular bioinformatics algorithm or workflow.

Computational aspects that are not specific to bioinformatics should preferably be covered by independent information-technology ontologies, such as, for example, the SWO (http://theswo.sourceforge.net) and the WSIO (http://wsio.org), the development of both of which is coordinated with the development of EDAM and the boundary concepts are referenced. EDAM agnostically links to multiple upper ontologies, allowing a plurality of future semantic-integration approaches. Some specific detailed concepts of data and methods are in focus of other ontologies, such as in case of the CDAO devoted to phylogenetics. In these cases EDAM excludes detailed concepts and instead refers to the boundary ones in the more specialized ontology. Different ontologies focussing on different semantic ‘axes’ than EDAM are clearly useful for enriching the annotations of tools or datasets, such as the SO, which may denote particular sequence features in focus of a tool or a dataset. In obvious candidates for such annotations, the relevant ontologies are referred to, such as in *Feature record* and *Feature prediction* concepts in EDAM pointing to *sequence_feature* in SO.

EDAM aims at being comprehensive for common concepts. Good coverage demands recurring input from the scientific community, in particular within specialized domains in which the core developers of EDAM lack expertise. For this purpose, a broader sustainable consortium should evolve in the future. EDAM will keep following the agile organic development model tested throughout the accomplished iterations. Thanks to the stable URIs and the deprecation mechanism, annotations remain valid with a release of a new version of the ontology. EDAM will continue being coordinated in harmony with related efforts, such as with SWO, WSIO, BioXSD and potentially others. The EDAM developers will continue improving EDAM, while being dependent on the community input and feedback from annotators, developers and users of bioinformatics tools. Additions and corrections can be suggested using a public issue tracker (http://www.ebi.ac.uk/panda/jira/browse/BMB). The EDAM team will continue providing support to the annotators and the application developers.
